# Granulomatosis with polyangiitis with obstructive pneumonia progressing to hypertrophic pachymeningitis: A case report

**DOI:** 10.1097/MD.0000000000024028

**Published:** 2021-01-22

**Authors:** Keigo Hayashi, Haruki Watanabe, Yuriko Yamamura, Yosuke Asano, Yu Katayama, Sumie Hiramatsu-Asano, Keiji Ohashi, Michiko Morishita, Mariko Narazaki, Yoshinori Matsumoto, Ken-Ei Sada, Jun Wada

**Affiliations:** Department of Nephrology, Rheumatology, Endocrinology and Metabolism, Okayama University Graduate School of Medicine, Dentistry and Pharmaceutical Sciences, Okayama, Japan.

**Keywords:** bronchial stenosis, granulomatosis with polyangiitis, hypertrophic pachymeningitis, rituximab

## Abstract

**Rationale:**

Bronchial involvement alone is a rare initial manifestation of granulomatosis with polyangiitis (GPA). Herein, we report a case of refractory GPA with obstructive pneumonia caused by bronchial involvement.

**Patient concerns:**

A 65-year-old man complained of a 2-week cough and fever.

**Diagnoses:**

Considering the presence of opacities and multiple consolidations in both lungs due to obstruction or stenosis on the bronchus, which did not respond to antibiotics, and proteinase-3-antineutrophil cytoplasmic autoantibody positivity, he was diagnosed with GPA. Positron emission tomography- computed tomography scan revealed no abnormal findings in the upper respiratory tract.

**Interventions:**

He was treated with prednisolone (PSL, 50 mg/d) and intravenous cyclophosphamide.

**Outcomes:**

His general and respiratory symptoms improved. However, 8 weeks after PSL treatment at 20 mg/d, he developed a relapse of vasculitis along with sinusitis and hypertrophic pachymeningitis. Hence, PSL treatment was resumed to 50 mg/d, and weekly administration of rituximab was initiated. Consequently, the symptoms gradually mitigated.

**Lessons:**

GPA with bronchial involvement is often intractable and requires careful follow-up, which should include upper respiratory tract and hypertrophic pachymeningitis assessment.

## 1 Introduction

Granulomatosis with polyangiitis (GPA) is one of the antineutrophil cytoplasmic autoantibody (ANCA)-associated vasculitides; it is a systemic, granulomatous, and necrotizing vasculitis that involves small- and medium-sized blood vessels and commonly affects the upper and lower respiratory tract.^[1]^

Severe bronchial stenosis (BS) is a rare initial respiratory involvement in GPA. Subglottic stenosis and tracheobronchial stenosis (TBS) distal to the glottis are reportedly observed in 12% to 23% of the GPA cases,^[2–5]^ whereas severe BS has been reported in only 4% of GPA cases with pulmonary manifestations.^[6]^ BS without the ear, nose, and throat (ENT) involvement has never been reported in GPA cases with BS. The tracheobronchial involvement is often intractable and requires multidisciplinary approaches, such as surgical intervention and local glucocorticoid injection.^[7–9]^ Although glucocorticoid combined with cyclophosphamide (CYC) or rituximab (RTX) is a recommended standard treatment for GPA, the appropriate treatment strategy for GPA with BS remains uncertain.

Herein, we report a patient with GPA who initially presented with only BS and experienced relapse with sinusitis and hypertrophic pachymeningitis (HP). The patient was then successfully treated with RTX.

## 2 Case presentation

**Figure 1:**
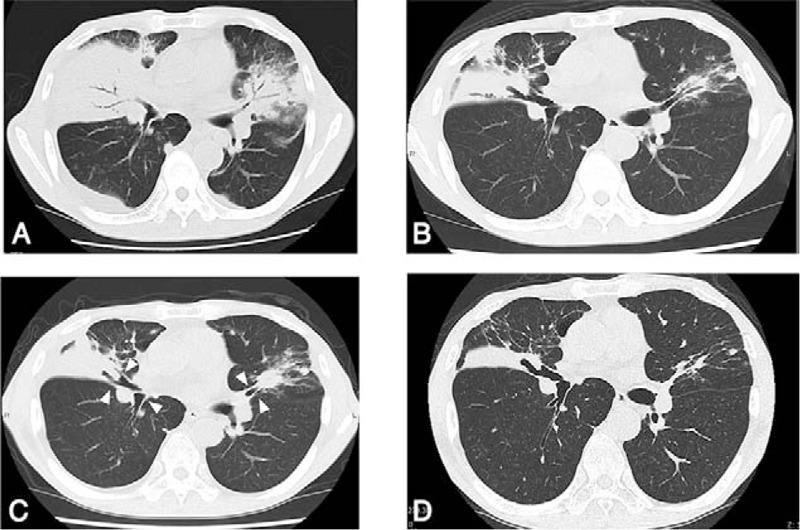
Computed tomography of the chest. (A) At the diagnosis. (B) One month later from the initiation of remission induction therapy. (C) At the time of relapse. (D) At 5 weeks after the initiation of re-remission induction therapy. The white arrowheads indicate the exacerbation of bronchial wall thickness.

A 65-year-old man was admitted to a community hospital with a 2-week history of cough and >38°C body temperature. Computed tomography (CT) of the lungs revealed that both lungs exhibited opacities and multiple consolidations (Fig. 1A). Initially, he was treated with antibiotics, but no signs of improvement were noted. Bronchoscopy was then conducted, which revealed that the bronchus of the right middle lobe was obstructed and that of the left upper lobe was red and fragile without active hemorrhage. Furthermore, proteinase-3 (PR3)-ANCA was positive; hence, he was transferred to our hospital at 4 weeks after the onset of symptoms.

Upon admission, he had persistent fever, weight loss, and cough but without symptoms involving ENT. Physical examination revealed the following: height, 168 cm; weight, 48 kg; body temperature, 37.6°C; and blood pressure, 114/64 mm Hg. No abnormal findings were detected in the head, neck, chest, musculoskeletal, and nervous systems. The laboratory findings were as follows: white blood cell count, 10460/μL (neutrophils: 94.4%); C-reactive protein level, 9.7 mg/dL; PR3-ANCA, 16 IU/mL (cutoff ≤ 2.00 IU/mL); and MPO-ANCA, negative. Urinalysis was normal, and the serum creatinine level was 0.52 mg/dL. According to the positron emission tomography-CT scan, ^18^F-fluorodeoxyglucose accumulated within both lungs, consistent with the opacities revealed in CT, but no abnormalities were found in ENT. CT-guided lung biopsy revealed necrotizing granulomatous inflammation with remarkable infiltrations of neutrophils and multinucleated giant cells. Subsequently, the patient was diagnosed with GPA according to the presence of chronic lower respiratory symptoms with multiple infiltrates of the lung and the PR3-ANCA positivity.

**Figure 2:**
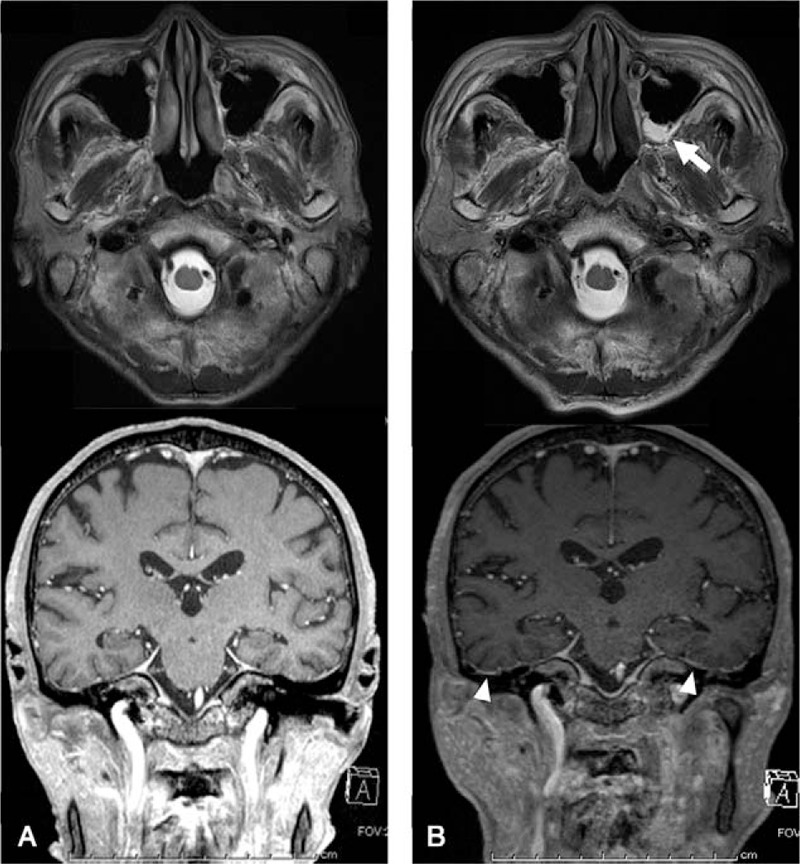
Magnetic resonance imaging of the head. (A) One month after the initiation of remission induction therapy. (B) At the time of relapse. The white arrow indicates the fluid in the left maxillary sinus, and the white arrowheads denote gadolinium enhancement of the hypertrophic dura mater.

After the patient received 50 mg/d of prednisolone (PSL) and intravenous CYC, his general and respiratory symptoms improved. Four weeks after treatment initiation, chest CT showed that the consolidations in the right upper lobe of the lung improved (Fig. 1B). In addition, enhanced magnetic resonance imaging (MRI) of the head revealed no findings indicative of sinusitis or HP 4 weeks after treatment initiation (Fig. 2A).

**Figure 3:**
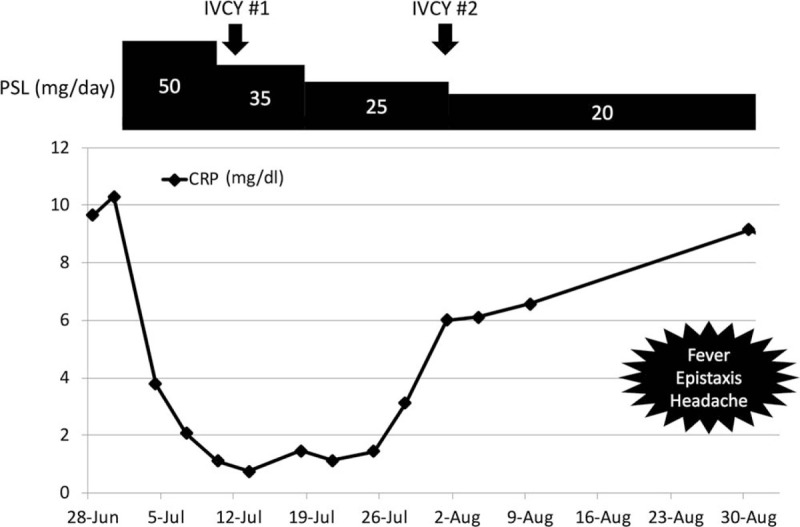
Clinical course from the presentation to the relapse. CRP = C-reactive protein, IVCY = intravenous cyclophosphamide, PSL = prednisolone.

However, 8 weeks after treatment with 20 mg/d (0.4 mg/kg/d) of PSL, the patient developed fever, epistaxis, and headache, with elevations of C-reactive protein levels to 9.2 mg/dL and PR3-ANCA levels to 24 IU/mL (Fig. 3). Chest CT detected that the left upper lobe of the lung consolidated and the bronchial wall thickened (Fig. 1C). Moreover, contrast-enhanced MRI revealed the presence of fluid in the left maxillary sinus and the abnormal dura thickening in the middle cranial fossa (Fig. 2B). Cerebrospinal fluid findings included the following; appearance: clear, opening pressure: 15 cm H_2_O, WBC count: 3 cells/μL (mononuclear cells), glucose level: 100 mg/dL, protein level: 105 mg/dL, IgG index: 0.58, mycobacterium; negative. There were no evidence of IgG4-related disease and sarcoidosis. Hence, the patient was diagnosed to develop a relapse of vasculitis with newly appeared sinusitis and HP. We then increased the dose of PSL to 50 mg/d and initiated weekly RTX (375 mg/m^2^/wk for 4 weeks) concomitantly. After receiving re-induction remission treatment, the patient's fever, epistaxis, and headache alleviated. Five weeks after this treatment, the opacities noted on the right middle lobe and left inferior lingular segment also improved (Fig. 1D). Ten months after the re-induction treatment, PSL was reduced to 2.5 mg/d without any additional treatment.

## 3 Discussion

To our knowledge, this is the first case of GPA presenting with only BS that resulted in obstructive pneumonia. The strength of the present case is that the absence of the upper airway disease at the presentation was confirmed by the imaging studies. BS alone is a rare manifestation as the first symptom of GPA. Generally, the upper respiratory tract is affected before the lower respiratory tract,^[10]^ and only 9% of patients reportedly did not have any other organ involvements other than the lungs.^[6]^ Among GPA cases with lung involvement, 4% reportedly had severe stenosis leading to atelectasis.^[6]^ As the limitation in the assessment of this case, MRI of the head was not conducted before the commencement of treatment. Therefore, HP might be present at the presentation despite no headache. It has been reported that HP accompanies in approximately 20% to 30% of GPA patients, and the pathogenic mechanism is thought to be the spreading of granulomatous tissue from the nasal, paranasal cavities, or orbit and contiguously invasion to the adjacent structures including meninges.^[11]^ A nationwide survey conducted in Japan showed that 34% of patients with HP were diagnosed with ANCA-associated vasculitides, and 9% of patients with IgG4-related disease.^[12]^ This patient lacked salivary and lacrimal gland enlargement, orbital disease, autoimmune pancreatitis, retroperitoneal fibrosis, and tubulointerstitial nephritis; therefore, we have excluded this differential diagnosis.

Previous reports showed tracheobronchial involvement in GPA is refractory and sometimes relapses with ENT involvement. It is reported that 62% of the patients with GPA exhibiting TBS and/or subglottic stenosis experienced relapses.^[5,9]^ Among the patients who experienced relapses with BS, 11% relapsed with ENT symptoms.^[9]^ In another study, rhinosinusal involvement associated with TBS.^[5]^ The present case relapsed with not only sinusitis but also HP despite the absence of ENT involvement at disease onset. Considering that HP is also refractory,^[13]^ ENT symptoms and HP need to be monitored carefully during the relapse of the lower respiratory symptoms.

The present case responded to RTX well in contrast to high-dose PSL and intravenous CYC. Girard et al described that RTX was more effective than CYC for treating GPA with BS.^[9]^ RTX is also reportedly effective for HP,^[14,15]^ and in our case, the PSL dosage could be reduced without relapses. It is possible that RTX is a preferred treatment option for GPA patients with BS.

In conclusion, healthcare providers need to consider not only lung cancer but also GPA despite the absence of upper airway symptoms. It also should be recognized that BS in GPA sometimes exacerbates with ENT involvement and HP during the clinical course. RTX might be a possible treatment option for GPA with BS and HP.

## Acknowledgments

We are thankful to all medical staff members in our department.

## Author contributions

**Conceptualization:** Keigo Hayashi, Haruki Watanabe.

**Data curation:** Keigo Hayashi, Haruki Watanabe.

**Investigation:** Keigo Hayashi, Haruki Watanabe, Yuriko Yamamura.

**Supervision:** Ken-Ei Sada, Jun Wada.

**Writing – original draft:** Keigo Hayashi, Haruki Watanabe.

**Writing – review & editing:** Haruki Watanabe, Yosuke Asano, Yu Katayama, Sumie Hiramatsu-Asano, Keiji Ohashi, Michiko Morishita, Mariko Narazaki, Yoshinori Matsumoto, Ken-Ei Sada.

## References

[1] Jennette JC Falk RJ Bacon PA 2012 revised International Chapel Hill consensus conference nomenclature of vasculitides. Arthritis Rheum 2013;65:1–1. 23045170 10.1002/art.37715

[2] Monach PA L25. Medical treatment of subglottic stenosis in granulomatosis with polyangiitis (Wegener's). Presse Med 2013;42:575–6. 23453209 10.1016/j.lpm.2013.01.025

[3] Langford CA Sneller MC Hallahan CW Clinical features and therapeutic management of subglottic stenosis in patients with Wegener's granulomatosis. Arthritis Rheum 1996;39:1754–60. 8843868 10.1002/art.1780391020

[4] Hoffman GS Kerr GS Leavitt RY Wegener granulomatosis: an analysis of 158 patients. Ann Intern Med 1992;116:488–98. 1739240 10.7326/0003-4819-116-6-488

[5] Marroquin-Fabian E Ruiz N Mena-Zuniga J Frequency, treatment, evolution, and factors associated with the presence of tracheobronchial stenoses in granulomatosis with polyangiitis. Retrospective analysis of a case series from a single respiratory referral center. Semin Arthritis Rheum 2019;48:714–9. 29891418 10.1016/j.semarthrit.2018.05.005

[6] Cordier JF Valeyre D Guillevin L Pulmonary Wegener's granulomatosis. A clinical and imaging study of 77 cases. Chest 1990;97:906–12. 2323259 10.1378/chest.97.4.906

[7] Hernandez-Rodriguez J Hoffman GS Koening CL Surgical interventions and local therapy for Wegener's granulomatosis. Curr Opin Rheumatol 2010;22:29–36. 19910793 10.1097/BOR.0b013e328333e9e9

[8] Gluth MB Shinners PA Kasperbauer JL Subglottic stenosis associated with Wegener's granulomatosis. Laryngoscope 2003;113:1304–7. 12897550 10.1097/00005537-200308000-00008

[9] Girard C Charles P Terrier B Tracheobronchial stenoses in granulomatosis with polyangiitis (Wegener's): a report on 26 cases. Medicine (Baltimore) 2015;94:e1088 doi: 10.1097/MD.0000000000001088. 26266344 10.1097/MD.0000000000001088 PMC4616693

[10] Reinhold-Keller E Beuge N Latza U An interdisciplinary approach to the care of patients with Wegener's granulomatosis: long-term outcome in 155 patients. Arthritis Rheum 2000;43:1021–32. 10817555 10.1002/1529-0131(200005)43:5<1021::AID-ANR10>3.0.CO;2-J

[11] Smoleńska Ż Masiak A Zdrojewski Z Hypertrophic pachymeningitis as an important neurological complication of granulomatosis with polyangiitis. Reumatologia 2018;56:399–405. 30647488 10.5114/reum.2018.80719 PMC6330683

[12] Yonekawa T Murai H Utsuki S A nationwide survey of hypertrophic pachymeningitis in Japan. J Neurol Neurosurg Psychiatry 2014;85:732–9. 24273222 10.1136/jnnp-2013-306410

[13] Ohashi K Morishita M Watanabe H Central diabetes insipidus in refractory antineutrophil cytoplasmic antibody-associated vasculitis. Intern Med 2017;56:2943–8. 28943556 10.2169/internalmedicine.8683-16 PMC5709644

[14] Sharma A Kumar S Wanchu A Successful treatment of hypertrophic pachymeningitis in refractory Wegener's granulomatosis with rituximab. Clin Rheumatol 2010;29:107–10. 19802640 10.1007/s10067-009-1291-z

[15] Shimojima Y Kishida D Hineno A Hypertrophic pachymeningitis is a characteristic manifestation of granulomatosis with polyangiitis: a retrospective study of anti-neutrophil cytoplasmic antibody-associated vasculitis. Int J Rheum Dis 2017;20:489–96. 28217942 10.1111/1756-185X.13046

